# Modifying the Health Star Rating nutrient profiling algorithm to account for ultra‐processing

**DOI:** 10.1111/1747-0080.12892

**Published:** 2024-07-10

**Authors:** Eden M. Barrett, Simone Pettigrew, Bruce Neal, Mike Rayner, Daisy H. Coyle, Alexandra Jones, Damian Maganja, Allison Gaines, Dariush Mozaffarian, Fraser Taylor, Nadine Ghammachi, Jason H. Y. Wu

**Affiliations:** ^1^ The George Institute for Global Health University of New South Wales Sydney New South Wales Australia; ^2^ School of Health Sciences, Faculty of Medicine and Health University of New South Wales Sydney New South Wales Australia; ^3^ Friedman School of Nutrition Science & Policy Food is Medicine Institute, Tufts University Boston Massachusetts USA; ^4^ Oxford Martin Programme on the Future of Food and Nuffield Department of Population Health University of Oxford Oxford UK; ^5^ Tufts School of Medicine and Division of Cardiology, Tufts Medical Center Boston Massachusetts USA; ^6^ School of Population Health, Faculty of Medicine and Health University of New South Wales Sydney New South Wales Australia

**Keywords:** food policy, front‐of‐pack label, Health Star Rating, NOVA, nutrient profiling, ultra‐processed

## Abstract

**Aim:**

To modify the Australian and New Zealand Health Star Rating to account for ultra‐processing and compare the alignment of the modified ratings with NOVA classifications and the current Australian Dietary Guidelines classifications of core (recommended foods) and discretionary (foods to limit).

**Methods:**

Data was cross‐sectionally analysed for 25 486 products. Four approaches were compared to the original Health Star Rating: (1) five ‘negative’ points added to ultra‐processed products (*modification 1; inclusion approach*); (2) ultra‐processed products restricted to a maximum of 3.0 Health Stars (*modification 2; capping approach*); (3 and 4) same approach used for modifications 1 and 2 but only applied to products that already exceeded 10 ‘negative’ points from existing Health Star Rating attributes (*modifications 3 and 4*, respectively; hybrid approaches). Alignment occurred when products (i) received <3.5 Health Stars and were NOVA group 4 (for NOVA comparison) or discretionary (for Dietary Guidelines comparison), or (ii) received ≥3.5 Health Stars and were NOVA groups 1–3 or core.

**Results:**

All Health Star Rating modifications resulted in greater alignment with NOVA (ranging from 69% to 88%) compared to the original Health Star Rating (66%). None of the modifications resulted in greater alignment to the Dietary Guidelines classifications overall (69% to 76%, compared with 77% for the original Health Star Rating), but alignment varied considerably by food category.

**Conclusions:**

If ultra‐processing were incorporated into the Australian and New Zealand Health Star Rating, consideration of ultra‐processing within the broader dietary guidance framework would be essential to ensure coherent dietary messaging to Australians.

## 1 INTRODUCTION

Non‐communicable diseases are responsible for nearly three‐quarters of deaths worldwide.^1^ Diet is a leading modifiable risk factor, accounting for 26% of all premature deaths globally in 2017.^2^ Emerging research indicates that the consumption of ultra‐processed foods, defined by the NOVA classification system, is a key component of unhealthy diets with associated increased risks of cardiovascular disease, metabolic syndrome, obesity and some cancers.^3^, ^4^

The NOVA classification system is a well‐evaluated system for distinguishing ultra‐processed foods and beverages.^5^ NOVA specifies four groups: (1) unprocessed or minimally processed foods, (2) processed culinary ingredients, (3) processed foods and (4) ultra‐processed foods.^5^ Ultra‐processed foods are characterised as industrially manufactured products made using a range of chemical and physical processing techniques.^5^ They typically contain food substances never or rarely used in home kitchens or cosmetic additives that transform the textural and sensory properties of foods, and often contain large amounts of refined starch, added salt, and added sugar.

Although ultra‐processed foods tend to be higher in unfavourable nutrients, studies suggest that the negative health impact of these products may extend beyond their nutrient profile.^6^, ^7^, ^8^ Some potential mechanisms include that food matrix disruption in ultra‐processing (i.e. the fractionation of foods and ingredients) may contribute to lower satiation and facilitate overeating,^9^ and consumption of industrial food additives and acellular nutrients have been linked to adverse effects in gut microbiota composition and function, increased intestinal permeability and subsequent inflammatory response.^8^, ^10^, ^11^ In many high‐income countries, ultra‐processed foods have been found to contribute to up to 30%–60% of total energy intake.^8^ Today, at least seven countries now recommend limiting the intake of ultra‐processed foods within their national dietary guidelines.^12^

The Health Star Rating is a voluntary front‐of‐pack nutrition labelling system endorsed by the Australian and New Zealand governments and implemented in 2014, aiming to assist consumers in making healthier packaged food choices.^13^ The Health Star Rating assigns a rating from 0.5 (least healthy) to 5 stars (most healthy) based on nutritional composition. The underlying algorithm currently grades energy, total sugar, sodium and saturated fat content as unfavourable factors, whereas protein, fibre, fruit, vegetable, nut and legume content are graded as favourable factors.

Although studies have shown that a healthy diet defined by the Health Star Rating is associated with a lower risk of weight gain,^14^ cardiovascular disease,^15^ and mortality,^15^ there may be room for improvement in the system. Concerns have been raised that it is too lenient on unhealthy products that might be ultra‐processed yet still receive high ratings due to its nutrient‐centric focus and lack of consideration for ultra‐processing.^16^, ^17^ Given the emerging evidence for the negative health effects of ultra‐processing independent of nutrient profile, considering ultra‐processing as a factor within the Health Star Rating algorithm might enhance its ability to distinguish between healthier and less healthy products. This consideration was put forward in stakeholder submissions to the 5‐year review of the Health Star Rating in 2017, but was not subject to detailed consideration or implemented further at that time.^18^ If the Health Star Rating algorithm were to be modified to account for ultra‐processing, care would need to be taken to ensure the resulting system remained aligned with other authoritative sources of dietary advice in Australia. This includes the Australian Dietary Guidelines, which are currently under review.^19^ Although the current Australian Dietary Guidelines (released in 2013) do not consider ultra‐processing, the review committee recently announced that level of processing, specifically ultra‐processing, is a very high priority area of this review.^20^

The aims of this investigation were to (i) explore methods for modifying the Health Star Rating algorithm to include ultra‐processing and (ii) identify the effects this would have on the rating of food and beverages within different categories. We compared the alignment between the Health Star Rating and modified Health Star Ratings with NOVA food processing classifications and the current Australian Dietary Guidelines classifications of core and discretionary.

## 2 METHODS

This cross‐sectional study has been designed and completed in accordance with the Strengthening the Reporting of Observational Studies in Epidemiology (STROBE) guidelines. The study contains no human or animal data and no ethics approval was required.

We used data from the George Institute for Global Health's Australian 2022 FoodSwitch Monitored data set (FoodSwitch).^21^ The 2022 FoodSwitch data set contains 35 645 barcoded products available for sale from five major supermarket retailers in Australia, representing over 90% sales‐weighted coverage of available barcoded products in Australia.^22^ The data set contains product‐specific nutrient and ingredient information obtained directly from packaging. Details of the data collection process are described elsewhere.^21^

Products within FoodSwitch are categorised based on a hierarchical system developed by the Global Food Monitoring Group.^23^ This system categorises products into major categories (e.g. bread and bakery products), categories (e.g. bread), and subcategories (e.g. pita bread). We excluded major categories not covered by the Health Star Rating system (vitamins and supplements, alcoholic beverages; *n* = 7134). We also excluded subcategories that are not permitted to display a Health Star Rating such as infant formula (*n* = 1000), and products without a nutrient information panel such as herbs and spices (*n* = 1916) except where a rating is automatically applied (e.g. plain bottled water and plain fruit and vegetables automatically receive a rating of 5.0). A further 109 products were excluded as they were missing nutrient data required to calculate a Health Star Rating. This left 25 486 individual products available for analysis.

The Health Star Rating was calculated for all eligible products as defined by Food Standards Australia New Zealand.^13^, ^24^, ^25^ Where information needed was not available from the packaging (specifically, per cent fruit, vegetable, nut and legume content and fibre content), it was provided by manufacturers or estimated using methods described previously.^26^ All products were categorised into one of the six Health Star Rating categories (non‐dairy beverages, dairy beverages, oils and spreads, cheese and processed cheese, all other dairy foods, and all other non‐dairy foods). Baseline (negative) points were calculated based on energy, saturated fat, total sugar and sodium content per 100 g; and modifying (positive) points based on per cent fruit, vegetable, nut and legume, protein, and fibre content per 100 g. A final score was calculated by subtracting the modifying points from the baseline points, which was then converted to a Health Star Rating based on the specific scoring matrix for each of the six categories.^13^

We used the inclusion of industrial food substances and/or cosmetic additives (markers of ultra‐processing) on products' ingredient lists as a proxy marker to identify ultra‐processed foods, following an adapted NOVA classification approach^25^ consistent with methods suggested by the researchers who developed the NOVA classification system.^5^ Briefly, markers of ultra‐processing included:

1. Any ingredient considered an anti‐caking, firming or glazing agent, colour, flavour, emulsifier, extract or thickener;
2. Any sweeteners other than sugar, maple syrup and honey (e.g. dextrose, fructose and maltitol);
3. Components that are inherent in whole foods but included as isolates (e.g. lactose, wheat gluten and triglycerides); and
4. Protein powders.

Vitamins, minerals and live cultures used as product fortifications or fermentation were not considered markers of ultra‐processing. Consistent with NOVA classification system developers, acids and acidity regulators, which can be added to products as preservatives but do not alter flavour or texture, were also not considered markers of ultra‐processing.^27^ A full list of ingredient list terms considered markers of ultra‐processing is included in Supporting Information S1: Table S1.

The Australian Dietary Guidelines differentiate between ‘discretionary’ and ‘non‐discretionary’ (referred to here as ‘core’) products.^28^ Discretionary foods are defined as energy‐dense and nutrient‐poor, and typically include items that are not essential for meeting the body's nutritional requirements.^28^, ^29^ Core foods are those that are included in the five recommended food groups: grain (cereal) foods; vegetables and legumes/beans; fruit; milk, yoghurt, cheese and/or alternatives; and lean meats and poultry, fish, eggs, nuts and seeds, and vegetarian alternatives. For the purposes of algorithm modification assessment, all products within the 2022 FoodSwitch data set were classified as core or discretionary by comparing them to the Australian Bureau of Statistics Discretionary Food List.^29^ The Discretionary Food List classifies foods as discretionary if specified or inferred in the Australian Dietary Guidelines and supporting documents as discretionary.^29^ As the Dietary Guidelines documents provide only limited examples of discretionary foods, for some categories, the Australian Bureau of Statistics applies additional nutrient criteria to define core and discretionary. For example, breakfast cereals with ≥35 g sugars per 100 g (with added fruit) or ≥30 g sugars per 100 g (without added fruit) are discretionary, all others are core. The nutrient criteria used were used in the modelling that supported the original guidelines development.^30^

**FIGURE 1:**
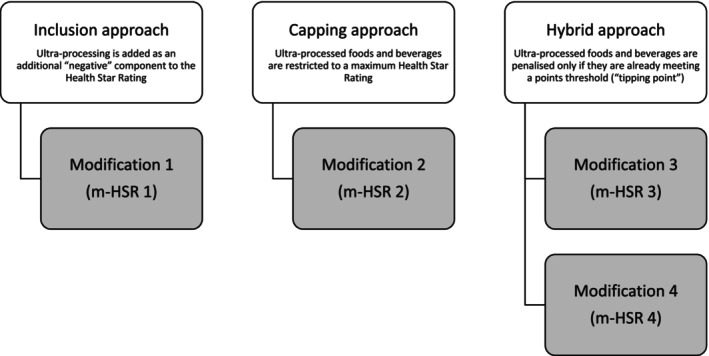
Four Health Star Rating (HSR) modifications (m‐HSR 1–4) nested within three general approaches: *inclusion* approach, *capping* approach and *hybrid* approach. Modification 1 (m‐HSR 1): Five additional baseline (negative) points added for ultra‐processed products across all six HSR categories; modification 2 (m‐HSR 2): All ultra‐processed products restricted to a maximum of 3.0 HSR regardless of their final HSR score; modification 3 (m‐HSR 3): As modification 1, but only applied to products that have 10 or more existing baseline points from energy/saturated fat/total sugar/sodium; modification 4 (m‐HSR 4): As modification 2, but only applied to products that have 10 or more existing baseline points from energy/saturated fat/total sugar/sodium.

To determine the potential impacts of a range of ultra‐processing‐related adjustments to the Health Star Rating algorithm, we tested four modifications nested within three general approaches: *inclusion*; *capping*; and *hybrid* (Figure 1). These approaches were chosen as they reflect current approaches in the algorithm and/or were used by the Technical Advisory Group in 2018 to model potential nutrient and food component changes to the algorithm in the five‐year review of the system.^31^

In modification 1 (m‐HSR 1), five additional baseline (negative) points were added for ultra‐processed foods across all six categories. In determining the number of baseline points to add, we assessed the addition of 2, 5 and 10 baseline points. Based on the range and distribution of baseline points assigned for existing components of the Health Star Rating, 5 points was considered reasonable to generate a proportional impact on the final score (resulting in an average of 1.5 star reduction across all ultra‐processed foods, compared to 3.0 star for 10 points and <1.0 star for 2 points added).

In modification 2 (m‐HSR 2), all ultra‐processed foods were restricted to a maximum Health Star Rating of 3.0 regardless of their final score. This meant that any ultra‐processed food assigned a rating of ≥3.5 was re‐classified to 3.0. In the absence of federal government‐endorsed Health Star Rating cut‐offs for healthy foods, the cut‐off of >3.0 for ‘healthy’ products was used as this has been used in previous research,^19^, ^25^ and is also incorporated in elements of various NSW jurisdiction guidelines.^32^, ^33^

Modification 3 (m‐HSR 3) was as modification 1, but only applied to products that have 10 or more existing baseline points. Based on the range and distribution of baseline points, 10 points was considered a reasonable tipping point to only target products ‘high’ in energy, saturated fat, total sugar and/or sodium. Similarly, modification 4 (m‐HSR 4) was as modification 2, but only applied to products that have 10 or more existing baseline points.

We examined the median rating and interquartile range of products according to the original Health Star Rating algorithm and each of the m‐HSR 1–4 algorithms overall and by major food category. We then examined the number of individual product shifts in HSR resulting from each modification.

We quantified the alignment of the original Health Star Rating and each m‐HSR to the NOVA level of processing and Australian Dietary Guidelines classifications of healthy food and beverages, respectively. Alignment was deemed to occur when products were identified as (i) Health Star Rating (or m‐HSR) of <3.5 and NOVA group 4 (for NOVA comparison) or discretionary (for Australian Dietary Guidelines comparison) and or (ii) Health Star Rating (or m‐HSR) of ≥3.5 and NOVA groups 1–3 or core. The number and proportion (*n*, %) of alignment overall and within each major category were quantified. All analyses were conducted in Stata version 18.

## 3 RESULTS

**FIGURE 2:**
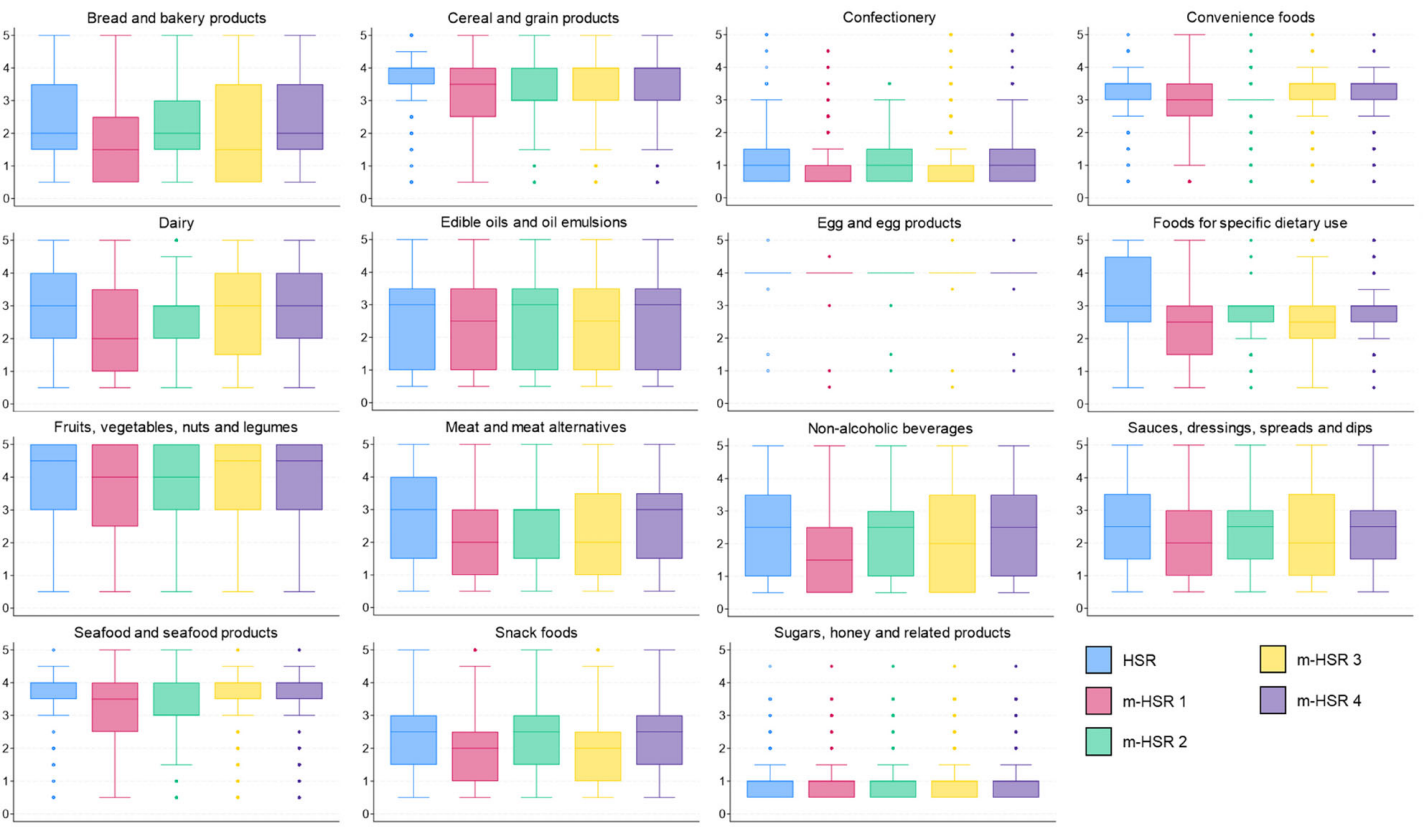
Health Star Rating (HSR) and m‐HSR 1–4 for 25 486 foods and beverages, shown by 15 major food categories. Standard box plots are shown, with horizontal lines representing the median HSR/m‐HSR, whiskers representing variance (interquartile range*1.5), and the small circles representing outliers. Modification 1 (m‐HSR 1): Five additional baseline (negative) points added for ultra‐processed products across all six HSR categories; modification 2 (m‐HSR 2): All ultra‐processed products restricted to a maximum of 3.0 HSR regardless of their final HSR score; modification 3 (m‐HSR 3): As modification 1, but only applied to products that have 10 or more existing baseline points from energy/saturated fat/total sugar/sodium; modification 4 (m‐HSR 4): As modification 2, but only applied to products that have 10 or more existing baseline points from energy/saturated fat/total sugar/sodium.

Among the 25 486 products included in the analysis, 16 371 (64%) were classified as ultra‐processed based on our ingredient‐based approach. Within major categories, this ranged from 5% (*n* = 4) of *egg and egg products*, to 96% (*n* = 1669) of *confectionery*. The median Health Star Rating across all products was 3.0 (1.5–4.0). This shifted to 2.0 (1.0–3.5) according to m‐HSR 1 and 2.5 (1.0–4.0) according to m‐HSR 3 but did not change substantially according to m‐HSR 2 or m‐HSR 4 (Supporting Information S1: Table S2). Within major food categories, none of the modifications resulted in a change in the median rating for *egg and egg products* or *sugar, honey and related products* (Figure 2, Supporting Information S1: Table S2). All other major categories had an overall decline in median rating of 0.5–1.0 stars when applying m‐HSR 1. When applying m‐HSR 2, four major categories had a decline in median rating of 0.5–1.0 stars (*cereal and grain products; convenience foods; fruit, vegetables, nuts and legumes;* and *seafood and seafood products*), and eight different major categories had a decline of 0.5–1.0 stars when applying m‐HSR 3 (*bread and bakery products; confectionery; edible oils and oil emulsions; foods for specific dietary uses; meat and meat alternatives; non‐alcoholic beverages; sauces, dressings, spreads and dips;* and *snack foods*). No major categories had a decline in median rating when applying m‐HSR 4. Overall, m‐HSR 1 resulted in the greatest number of ultra‐processed foods shifting to a lower rating (*n* = 13 543; 83%). By comparison, the number of ultra‐processed foods receiving a lower rating from m‐HSR 2, m‐HSR 3 and m‐HSR 4 were 5620 (34%), 7393 (45%) and 1030 (6%), respectively.

As expected, all modifications (m‐HSR 1–4) resulted in greater alignment with the NOVA classification system compared to the current Health Star Rating (Table 1). M‐HSR 1 and 2 resulted in the greatest increases in alignment (from 66% to 81% and 88%, respectively), whereas the two‐hybrid approaches (m‐HSR 3 and 4) saw modest increases (69% and 70%, respectively). The major food categories with the greatest gain in alignment to the NOVA classification system when applying m‐HSR 1 and 2 compared to the current Health Star Rating were *cereal and grain products* (61% to 86% and 95%, respectively) *convenience foods* (42% to 86% and 96%, respectively)*, foods for specific dietary use* (53% to 87% and 97%, respectively), and *meat and meat alternatives* (53% to 77% and 91%, respectively). Alignment was also substantially higher for *foods for specific dietary uses* when applying m‐HSR 3 and 4 (from 53% to 78% and 80%, respectively).

**TABLE 1:** Number (*n*) and percentage (%) of products aligned with NOVA level of processing^a^ and Australian Dietary Guidelines classifications^b^ overall and within each major category, according to the Health Star Rating (HSR) and m‐HSR 1–4.^c^

		Alignment with NOVA^a^						Alignment with Australian Dietary Guidelines^b^				
Major food category	*N*	HSR	m‐HSR 1	m‐HSR 2	m‐HSR 3	m‐HSR 4		HSR	m‐HSR 1	m‐HSR 2	m‐HSR 3	m‐HSR 4
Overall	25 486	16 758 (66%)	20 565 (81%)	22 378 (88%)	17 637 (69%)	17 788 (70%)		19 606 (77%)	18 391 (72%)	17 524 (69%)	19 495 (76%)	19 410 (76%)
Bread and bakery products	3002	2140 (71%)	2541 (85%)	2819 (94%)	2232 (74%)	2244 (75%)		2527 (84%)	2332 (78%)	2100 (70%)	2535 (84%)	2535 (84%)
Cereal and grain products	1965	1191 (61%)	1684 (86%)	1870 (95%)	1359 (69%)	1369 (70%)		1562 (79%)	1077 (55%)	911 (46%)	1414 (72%)	1404 (71%)
Confectionery	1737	1611 (93%)	1661 (96%)	1672 (96%)	1614 (93%)	1614 (93%)		1673 (96%)	1723 (99%)	1734 (100%)	1676 (96%)	1676 (96%)
Convenience foods	1787	757 (42%)	1530 (86%)	1720 (96%)	797 (45%)	797 (45%)		1334 (75%)	591 (33%)	409 (23%)	1302 (73%)	1302 (73%)
Dairy	3169	1930 (61%)	2442 (77%)	2616 (83%)	1985 (63%)	2026 (64%)		2033 (64%)	1671 (53%)	1569 (50%)	1982 (63%)	1941 (61%)
Edible oils and oil emulsions	517	285 (55%)	315 (61%)	323 (62%)	315 (61%)	322 (62%)		374 (72%)	344 (67%)	336 (65%)	344 (67%)	337 (65%)
Egg and egg products	89	87 (98%)	88 (99%)	89 (100%)	87 (98%)	87 (98%)		87 (98%)	86 (97%)	85 (96%)	87 (98%)	87 (98%)
Foods for specific dietary use	403	213 (53%)	351 (87%)	390 (97%)	314 (78%)	323 (80%)		234 (58%)	338 (84%)	371 (92%)	309 (77%)	318 (79%)
Fruit, vegetables, nuts and legumes	3770	2826 (75%)	2957 (78%)	3248 (86%)	2857 (76%)	2898 (77%)		3223 (85%)	3182 (84%)	3021 (80%)	3206 (85%)	3167 (84%)
Meat and meat alternatives	1701	904 (53%)	1303 (77%)	1551 (91%)	1085 (64%)	1089 (64%)		1279 (75%)	1286 (76%)	1142 (67%)	1270 (75%)	1266 (74%)
Non‐alcoholic beverages	2069	1313 (63%)	1715 (83%)	1788 (86%)	1330 (64%)	1330 (64%)		1192 (58%)	1398 (68%)	1415 (68%)	1175 (57%)	1175 (57%)
Sauces, dressings, spreads and dips	2801	1982 (71%)	2169 (77%)	2285 (82%)	1997 (71%)	2014 (72%)		2167 (77%)	2318 (83%)	2388 (85%)	2158 (77%)	2155 (77%)
Seafood and seafood products	806	443 (55%)	554 (69%)	712 (88%)	461 (57%)	461 (57%)		576 (71%)	521 (65%)	481 (60%)	564 (70%)	564 (70%)
Snack foods	1244	914 (73%)	1076 (87%)	1116 (90%)	1042 (84%)	1052 (85%)		942 (76%)	1104 (89%)	1142 (92%)	1070 (86%)	1080 (87%)
Sugar, honey and related products	426	162 (38%)	179 (42%)	179 (42%)	162 (38%)	162 (38%)		403 (95%)	420 (99%)	420 (99%)	403 (95%)	403 (95%)

^a^ Alignment is defined as products that are ultra‐processed (NOVA group 4) and HSR/m‐HSR <3.5 or non‐ultra‐processed (NOVA groups 1–3) and HSR/m‐HSR ≥3.5.

^b^ Alignment is defined as products that are discretionary and HSR/m‐HSR <3.5 or core and HSR/m‐HSR ≥3.5.

^c^ Modification 1 (m‐HSR 1): Five additional baseline (negative) points added for ultra‐processed products across all six HSR categories; modification 2 (m‐HSR 2): All ultra‐processed products restricted to a maximum of 3.0 HSR regardless of their final HSR score; modification 3 (m‐HSR 3): As modification 1, but only applied to products that have 10 or more existing baseline points from energy/saturated fat/total sugar/sodium; modification 4 (m‐HSR 4): As modification 2, but only applied to products that have 10 or more existing baseline points from energy/saturated fat/total sugar/sodium.

None of the modifications resulted in overall greater alignment with the Australian Dietary Guidelines classifications (Table 1). M‐HSR 1 and 2 resulted in decreased alignment when compared to the current Health Star Rating (from 77% to 72% and 69%, respectively), whereas application of m‐HSR 3 and 4 did not meaningfully change alignment (from 77% to 77% and 76%, respectively). However, there was considerable variation among major food categories. Alignment within *foods for specific dietary use* and *snack foods* categories was greater across all modifications compared to the current Health Star Rating (from 58% to 77%–92%; and from 76% to 86%–92%; respectively). m‐HSR 1 and 2 also resulted in greater alignment within *non‐alcoholic beverages* (from 58% to 68% for both). Alignment within *cereals and grain products* was lower across all modifications compared to the current Health Star Rating (from 79% to 46%–72%); and for m‐HSR 1 and 2, substantially lower within *convenience foods* (from 75% to 33% and 23%, respectively).

## 4 DISCUSSION

Two modifications to the Health Star Rating algorithm to account for ultra‐processing that added penalties or capped scores for all ultra‐processed foods (m‐HSR 1 and m‐HSR 2) were effective in penalising these foods. Both modifications resulted in greater misalignment to the Australian Dietary Guidelines overall, although this differed by food category, with some food categories demonstrating greater alignment following modifications. Hybrid approaches that made lesser modifications to the algorithm (m‐HSR 3 and m‐HSR 4) retained better broad alignment to the Australian Dietary Guidelines, but only modestly penalised ultra‐processed foods.

The application of the ‘inclusion’ approach (m‐HSR 1) proved most effective in penalising ultra‐processed foods and as anticipated also led to a substantial improvement in alignment with the NOVA system. One benefit of this approach is that it enhanced differentiation between ultra‐processed and non‐ultra‐processed versions of foods with favourable nutrient profiles (e.g. non‐ultra‐processed wholemeal bread received 4.0–4.5 m‐HSR and ultra‐processed wholemeal bread received 3.5–4.0 m‐HSR), whereas still rating these foods substantially higher than ultra‐processed foods that were *also* penalised already for having a poor nutrient profile (e.g. all brownies received ≤2.0 m‐HSR). However, it did reduce alignment with the Australian Dietary Guidelines within some food categories, particularly *cereal and grain products, convenience products* and *dairy*. Within these categories, commonly affected products included instant oatmeal, frozen/chilled prepackaged meals and plant‐based milk. Reducing the Health Star Rating of these foods could potentially confuse consumers because they are encouraged within the current Australian Dietary Guidelines. Therefore, further research is needed to specifically examine the health effects of these core ultra‐processed products to determine whether these products, despite being ultra‐processed, could still be suitable components of a healthy diet.

For other categories, such as *foods for specific dietary uses, snack foods* and *non‐alcoholic beverages*, alignment to the Australian Dietary Guidelines improved with application of m‐HSR 1, because discretionary products such as protein balls with high ratings due to added fibre and protein, and diet soft drinks with high ratings due to being low in energy and sugar, were reduced to m‐HSR <3.5. These changes would address a perceived flaw of the current nutrient‐centric Health Star Rating approach that allows manufacturers to use additives such as protein isolates, added fibres and non‐nutritive sweeteners to produce a ‘health halo’ for their products and boost the rating of otherwise highly processed, discretionary foods.^34^

The hybrid inclusion approach (m‐HSR 3), where the additional baseline points as per m‐HSR 1 were only added to ultra‐processed foods already accumulating 10 or more baseline points, resulted in around half the ultra‐processed foods being penalised but retained better alignment to the Australian Dietary Guidelines overall. The hybrid inclusion approach is a more conservative inclusion of ultra‐processing and would not impact most foods that are currently encouraged within the Australian Dietary Guidelines. This is because unlike some of the ultra‐processed foods affected by m‐HSR 1, those with 10 or more baseline points were already likely to meet the definition of discretionary, such as packaged pastries, iced confectionery, and soft drinks. However, it will not achieve the same differentiation between ultra‐processed and non‐ultra‐processed versions of foods, which could be one of the main purposes of introducing ultra‐processing as a component of the Health Star Rating.

The capping approach (m‐HSR 2), included for its simplicity in implementation, selectively penalised ultra‐processed foods with a high Health Star Rating (≥3.5). Because this approach did not further penalise nutrient‐poor ultra‐processed foods, it could cause confusion because foods with better nutrient profiles could receive the same rating as those with worse nutrient profiles. This is evidenced by the decline in median rating across the major categories of *cereals and grains, convenience foods, fruit, vegetables, nuts and legumes,* and *seafood*, where nutrient profiles tend to be more favourable. Consequently, this method led to the greatest reduction in overall alignment with the Australian Dietary Guidelines. However, similarly to m‐HSR 1, alignment for categories containing fewer core foods such as *foods for specific dietary uses, snack foods* and *non‐alcoholic beverages*, was improved. The hybrid version of this approach (m‐HSR 4) had minimal impact on the rating of products because it only targeted ultra‐processed foods that had both a rating of ≥3.5 and 10 or more baseline points. Very few of these products existed as having 10 or more baseline points typically led to a rating lower than 3.5.

We used alignment to the current Australian Dietary Guidelines classifications of core and discretionary as a measure of validity because the Guidelines are the current authoritative advice on dietary patterns in Australia. Although the Health Star Rating provides guidance on individual foods rather than dietary patterns, alignment through the provision of complementary guidance is an important consideration when implementing changes to food policy.^19^ Given the Australian Dietary Guidelines do not currently consider the concept of ultra‐processing, it was not surprising that the more aggressive penalisation approaches tested here resulted in increased misalignment in certain food categories. Our previous study demonstrates that approximately 40% of core foods as per the Australian Dietary Guidelines are ultra‐processed,^35^ with similar findings reported in other unpublished research.^36^ Our findings suggest that ensuring coherence with the existing dietary guidelines could be a challenge in incorporating ultra‐processing into the Health Star Rating. However, the Australian Dietary Guidelines were last released in 2013 and are currently in the process of being reviewed. The Australian Dietary Guidelines review committee recently announced that level of processing, specifically ultra‐processing, is a very high priority area of this review,^20^ a result of extensive public consultation. Therefore, it seems likely that the extent of difference in alignment found here with the current dietary guidelines could be reduced if the revised dietary guidelines also incorporate ultra‐processing to further differentiate foods within food groups.

Our study is not the first to explore incorporating ultra‐processing into food classification systems. The Siga classification scheme combines the 4 NOVA group classifications with additional subgroups (applied only within ultra‐processed and processed food classifications) that consider elements including contents of added sugar, salt and fat and the number and type of markers of ultra‐processing.^37^ A similar ‘top‐down’ (i.e. starting with processing and then nutritional composition) food classification system has been proposed by researchers for use in Australia.^38^ The Pan American Health Organisation's Nutrient Profile Model employs nutrient‐criteria, but only applies the criteria to processed and ultra‐processed products.^39^ The Food Compass incorporates information on nutrients, food ingredients and processing characteristics including NOVA categorisation.^40^ Unlike most of these approaches, the current study could be considered to have used ‘bottom‐up’ approaches because we classified foods starting from nutritional composition *and then* adjusted ratings for level of processing. This means that some ultra‐processed foods are still able to score a moderate or high rating via their favourable nutrient composition. The Australian and New Zealand Governments have committed to using the Health Star Rating, which necessitated starting with the current nutrient thresholds to ensure the feasibility of our approaches within the existing framework of the algorithm. However, future research comparing the product ratings and alignment to existing frameworks achieved from ‘top‐down’ approaches could be insightful. Another approach that may alleviate the complexity of attempting to incorporate both dimensions into a single metric is to provide the level of processing information alongside but separate from a nutrient‐based score. Although it could be expected that this approach may cause confusion for consumers in cases where the two classifications are in stark contrast, a recent study of a modified European Nutri‐score found this approach facilitated consumer understanding of both metrics independently.^41^

As a relatively new concept in nutrition science, the definition of ultra‐processed is continuing to be refined, which will also influence how it could be incorporated in food policies. Evidence is beginning to emerge to suggest that various types of ultra‐processed foods may contribute differently to the risk of disease.^42^, ^43^ A recent study of over 250 000 participants found that ultra‐processed animal‐based products and artificially‐ and sugar‐sweetened beverages were associated with increased risk of cancer and cardiovascular disease, but ultra‐processed breads and cereals were associated with marginally lowered risk.^42^ If confirmed in future studies, such findings would lend further weight to the hybrid approaches explored here (e.g. m‐HSR 3) or similar approaches that recognise nuance beyond ultra‐processing (as currently defined) as relevant to health harms.

The strengths of study include the use of data from over 25 000 products that are representative of the contemporary Australian packaged food supply and our systematic process of categorising individual products according to an adapted NOVA classification system. However, some limitations in our data also exist. In Australia, NIPs are not required to include fruit, vegetable, nut and legume content or fibre content. As a result, missing values were estimated using ingredient lists, food composition databases, and other available sources. Although our systematic method to identify ultra‐processed foods aligns with the recommended approach,^5^ there is debate regarding whether an ingredients list can accurately capture processing methods undertaken. However, given the complexity of applying NOVA classifications to individual products in large data sets, we consider our approach appropriate. Finally, the Health Star Rating cut‐off of ≥3.5 used across analyses is not universally accepted as appropriate to distinguish healthy and unhealthy ratings.^34^ Other researchers in Australia have suggested the use of a more stringent cut‐off (≥2.5).^34^, ^44^ Use of a different cut‐off such as ≥2.5 could result in a different degree of alignment than found here for each modification.

Our analysis of 25 486 foods and beverages available in Australia demonstrates the potential impacts of modifying the HSR algorithm using different approaches to account for the level of processing of foods and beverages. Modifications that had the biggest impact on penalising ultra‐processed foods also led to greater misalignment to the current Australian Dietary Guidelines classifications for some food categories. In conclusion, our study demonstrates that it is feasible to incorporate ultra‐processing into Australia's current Health Star Rating front‐of‐pack labelling system through several mechanisms and in doing so could better reflect the emerging evidence base on the health impacts of ultra‐processing. However, to ensure coherent dietary messaging to Australians, consideration of ultra‐processing within the broader dietary guidance framework would also be essential.

## AUTHOR CONTRIBUTIONS

Conceptualization: EMB, SP, MR and JHYW. Data curation: EMB and NG. Formal analysis: EMB. Funding acquisition: SP, JHYW, BN, MR, AJ, DMo and FT. Investigation: EMB. Methodology: EMB, SP, MR and JHYW. Project administration: EMB and NG. Supervision: EMB, SP and JHYW. Writing—original draft: EMB and JHYW. Writing—reviewing and editing: SP, AG, BN, DHC, DMa, AJ, MR, DMo and FT. EMB had primary responsibility for the final content. All authors are in agreement with the manuscript and declare that the content has not been published elsewhere.

## FUNDING INFORMATION

This work was supported by a National Health and Medical Research Council. The opinions, analysis and conclusions in this paper are those of the authors and should not be attributed to the NHMRC. S. Pettigrew, M. Rayner, B. Neal, A. Jones, D. Mozaffarian, F. Taylor and J. H. Y. Wu are all named CIs or AIs.

## CONFLICT OF INTEREST STATEMENT

Financial interests: D. Mozaffarian reports research funding from the National Institutes of Health, the Gates Foundation, The Rockefeller Foundation, Vail Innovative Global Research and the Kaiser Permanente Fund at East Bay Community Foundation; personal fees from Acasti Pharma and Barilla; scientific advisory board, Beren Therapeutics, Brightseed, Calibrate, Elysium Health, Filtricine, HumanCo, Instacart, January Inc., Perfect Day, Tiny Organics and (ended) Day Two, Discern Dx and Season Health; stock ownership in Calibrate and HumanCo; and chapter royalties from UpToDate. F. Taylor is the Managing Director of FoodSwitch—a data‐technology platform owned by TGI and run as a social enterprise. It receives revenue from industry, government and advocacy groups/NPFs for the following services: Independent data analyses by TGI using the FoodSwitch data; providing secure access to FoodSwitch data for third parties to conduct their own analyses; licencing of its data to third parties. The work is transactional (fee for service) and FoodSwitch/TGI does not endorse third‐party products or claims. Non‐financial interests: D. Mozaffarian is part of the Tufts University team that developed the Food Compass. M. Rayner, while a researcher working for the University of Oxford, has been closely involved in the development of the UK FSA/Ofcom, WHO‐Euro and other WHO regional nutrient profile models. S. Pettigrew has unpaid roles on nutrition‐related government committees including the Health Star Rating Advisory Committee and the Healthy Food Partnership Implementation Working Group.

## Supporting information


**Data S1:** Supporting Information.

## Data Availability

The data that support the findings of this study are available from FoodSwitch, but restrictions apply to the availability of these data, which were used under licence for the current study and so are not publicly available.
