# Supplementary material for: Modifying the Health Star Rating nutrient profiling algorithm to account for ultra‐processing
Source: Nutr Diet. 2024 Jul 10;82(1):53–63. doi: 10.1111/1747-0080.12892 (PMC11795220; doi:10.1111/1747-0080.12892)
Supplement: Supplementary file 1 — Data S1: Supporting Information. [file NDI-82-53-s001.docx]

**Supplementary Materials**

**Table S1. Ingredients treated as markers of ultra-processed foods (i.e., cosmetic additives and industrial food substances) when present on nutrition information panel ingredient lists**

| Agar | Gums (Arabic, guar, vegetable, xanthan) |
| --- | --- |
| Agent (anti-caking, de-foaming, firming, glazing) | Humectant |
| Aspartame | Hydrogenated/interesterified oil |
| Binder | Improver |
| Beta carotene | Inositol |
| Caffeine | Invert sugar |
| Carbon dioxide | Lactose |
| Carrageenan | Maltitol |
| Casein | Maltose |
| Charcoal | Mannitol |
| Coffee essence | Menthol |
| Colour/Color | Modified (maize/potato) starch |
| Confectioners glaze | Monoglyceride |
| Corn syrup (incl. high fructose) | Oligosaccharides |
| Dextrose | Pectin |
| Dextrin | Propane |
| Disodium guanosinemonophosphate | Propellant |
| Emulsifier | Protein powder/isolate (including hydrolysed/hydrolyzed) |
| Emulsifying salt | Sodium nitrate |
| Erythritol | Sodium nitrite |
| Esters | Sorbitol |
| Extract | Soy lecithin |
| Flavour/Flavor | Stabiliser/stabilizer |
| Food additive (including processing aid, sequestrants 452, 385, etc.) | Sucralose |
| Fructose | Sucrose fatty acid esters |
| Fruit juice concentrate | Sweetener (including acesulphame potassium (950) and aspartame (951) etc.) |
| Glucose | Thickener |
| Glucuronolactone | Trehalose |
| Gluten | Triglycerides |
| Glycerine | Xylitol |
| Glycerol |  |

**Table S2. Number and percent (%) of ultra-processed products and median (interquartile range) HSR according to current HSR and modified HSRs (m-HSR 1-4)^a^, overall and by major food categories**

| **Category (n)** | **Ultra-processed n (%)** | **HSR** | **m-HSR 1** | **m-HSR 2** | **m-HSR 3** | **m-HSR 4** |
| --- | --- | --- | --- | --- | --- | --- |
| Overall | 16,371 (64%) | 3.0 (1.5-4.0) | 2.0 (1.0-3.5) | 3.0 (1.5-3.0) | 2.5 (1.0-4.0) | 3.0 (1.5-4.0) |
| Bread and bakery products | 2586 (86%) | 2.0 (1.5-3.5) | 1.5 (0.5-2.5) | 2.0 (1.5-3.0) | 1.5 (0.5-3.5) | 2.0 (1.5-3.5) |
| Cereal and grain products | 996 (51%) | 4.0 (3.5-4.0) | 3.5 (2.5-4.0) | 3.0 (3.0-4.0) | 4.0 (3.0-4.0) | 4.0 (3.0-4.0) |
| Confectionery | 1669 (96%) | 1.0 (0.5-1.5) | 0.5 (0.5-1.0) | 1.0 (0.5-1.5) | 0.5 (0.5-1.0) | 1.0 (0.5-1.5) |
| Convenience foods | 1445 (81%) | 3.5 (3.0-3.5) | 3.0 (2.5-3.5) | 3.0 (3.0-3.0) | 3.5 (3.0-3.5) | 3.5 (3.0-3.5) |
| Dairy | 1851 (58%) | 3.0 (2.0-4.0) | 2.0 (1.0-3.5) | 3.0 (2.0-3.0) | 3.0 (1.5-4.0) | 3.0 (2.0-4.0) |
| Edible oils and oil emulsions | 124 (24%) | 3.0 (1.0-3.5) | 2.5 (1.0-3.5) | 3.0 (1.0-3.5) | 2.5 (1.0-3.5) | 3.0 (1.0-3.5) |
| Egg and egg products | 4 (5%) | 4.0 (4.0-4.0) | 4.0 (4.0-4.0) | 4.0 (4.0-4.0) | 4.0 (4.0-4.0) | 4.0 (4.0-4.0) |
| Foods for specific dietary use | 386 (96%) | 3.0 (2.5-4.5) | 2.5 (1.5-3.0) | 3.0 (2.5-3.0) | 2.5 (2.0-3.0) | 3.0 (2.5-3.0) |
| Fruits, vegetables, nuts, and legumes | 948 (25%) | 4.5 (3.0-5.0) | 4.0 (2.5-5.0) | 4.0 (3.0-5.0) | 4.5 (3.0-5.0) | 4.5 (3.0-5.0) |
| Meat and meat alternatives | 1435 (84%) | 3.0 (1.5-4.0) | 2.0 (1.0-3.0) | 3.0 (1.5-3.0) | 2.0 (1.0-3.5) | 3.0 (1.5-3.5) |
| Non-alcoholic beverages | 1550 (75%) | 2.5 (1.0-3.5) | 1.5 (0.5-2.5) | 2.5 (1.0-3.0) | 2.0 (0.5-3.5) | 2.5 (1.0-3.5) |
| Sauces, dressings, spreads, and dips | 1857 (66%) | 2.5 (1.5-3.5) | 2.0 (1.0-3.0) | 2.5 (1.5-3.0) | 2.0 (1.0-3.5) | 2.5 (1.5-3.0) |
| Seafood and seafood products | 332 (41%) | 4.0 (3.5-4.0) | 3.5 (2.5-4.0) | 3.0 (3.0-4.0) | 4.0 (3.5-4.0) | 4.0 (3.5-4.0) |
| Snack foods | 1015 (82%) | 2.5 (1.5-3.0) | 2.0 (1.0-2.5) | 2.5 (1.5-3.0) | 2.0 (1.0-2.5) | 2.5 (1.5-3.0) |
| Sugars, honey, and related products | 173 (41%) | 1.0 (0.5-1.0) | 1.0 (0.5-1.0) | 1.0 (0.5-1.0) | 1.0 (0.5-1.0) | 1.0 (0.5-1.0) |

1. Modification 1 (m-HSR 1): Five additional baseline (“negative”) points added for ultra-processed products across all six HSR categories; modification 2 (m-HSR 2): All ultra-processed products restricted to a maximum of 3.0 HSR regardless of their final HSR score; modification 3 (m-HSR 3): As modification 1, but only applied to products that have 10 or more existing baseline points from energy/saturated fat/total sugar/sodium; modification 4 (m-HSR 4): As modification 2, but only applied to products that have 10 or more existing baseline points from energy/saturated fat/total sugar/sodium.
